# Study of extravisual resting-state networks in pituitary adenoma patients with vision restoration

**DOI:** 10.1186/s12868-022-00701-3

**Published:** 2022-03-17

**Authors:** Fuyu Wang, Tao Zhou, Peng Wang, Ze Li, Xianghui Meng, Jinli Jiang

**Affiliations:** Department of Neurosurgery, The First Medical Center, Chinese PLA General Hospital, Beijing, China

**Keywords:** Cross-modal plasticity, Default mode network, Resting-state functional magnetic resonance imaging, Salience network, Visual improvement

## Abstract

**Background:**

Pituitary adenoma (PA) may compress the optic apparatus, resulting in impaired vision. Some patients can experience improved vision rapidly after surgery. During the early period after surgery, however, the change in neurofunction in the extravisual cortex and higher cognitive cortex has yet to be explored.

**Objective:**

Our study focused on the changes in the extravisual resting-state networks in patients with PA after vision restoration.

**Methods:**

We recruited 14 patients with PA who experienced visual improvement after surgery. The functional connectivity (FC) of 6 seeds [auditory cortex (A1), Broca’s area, posterior cingulate cortex (PCC) for the default mode network (DMN), right caudal anterior cingulate cortex for the salience network (SN) and left dorsolateral prefrontal cortex for the executive control network (ECN)] were evaluated. A paired t test was conducted to identify the differences between two groups of patients.

**Results:**

Compared with their preoperative counterparts, patients with PA with improved vision exhibited decreased FC with the right A1 in the left insula lobule, right middle temporal gyrus and left postcentral gyrus and increased FC in the right paracentral lobule; decreased FC with the Broca in the left middle temporal gyrus and increased FC in the left insula lobule and right thalamus; decreased FC with the DMN in the right declive and right precuneus; increased FC in right Brodmann area 17, the left cuneus and the right posterior cingulate; decreased FC with the ECN in the right posterior cingulate, right angular and right precuneus; decreased FC with the SN in the right middle temporal gyrus, right hippocampus, and right precuneus; and increased FC in the right fusiform gyrus, the left lingual gyrus and right Brodmann area 19.

**Conclusions:**

Vision restoration may cause a response of cross-modal plasticity and multisensory systems related to A1 and the Broca. The DMN and SN may be involved in top-down control of the subareas within the visual cortex. The precuneus may be involved in the DMN, ECN and SN simultaneously.

## Background

Experience-dependent plasticity gives individuals the ability to shape the visual cortex and maintain its normal function. It is present not only in the developing visual cortex but also in the adult visual cortex [1]. Therapeutic interventions can also trigger plastic changes in the aging visual cortex by restoring vision. Cataract surgery induced use-dependent structural plasticity in the secondary visual cortex (V2) by restoring impaired vision, and activity-dependent cortical plasticity was preserved in the aging visual cortex [2]. The responses to motion in the dorsal visual pathway decreased bilaterally and those to faces in the right ventral visual pathway increased after visual restoration [3]. In patients with Pituitary adenoma (PA) who had improved vision at approximately 3 months after the operation, the results showed that regional homogeneity (ReHo) decreased or increased within the visual cortex [4]. Most of these studies focused on neuroplasticity changes within the visual cortex; visual restoration can lead to plasticity in the subareas of the multisensory and multimodal systems beyond the visual cortex [4].

Cross-modal plasticity refers to the capacity to develop structural and functional changes to execute other intact senses when sensory input is lost. For compensation, the brain develops or strengthens corticocortical or subcorticocortical connections between the deprived and intact sensory regions [5, 6]. The loss of vision from birth triggers many compensatory plastic changes. The occipital cortex can be recruited by other nonvisual inputs because of the cross-modal reorganization of the brain [7]. The primary visual cortex (V1) was found to process auditory signals in persons with low-vision and those who suffered from blindness [7–11]. The visual cortex in humans with congenital blindness can control speech function and semantic processing [12, 13]. The auditory cortex can also process vision in deaf adults [14–16]. Although many studies have shown cross-modal plasticity in the loss of vision input, little has been reported about this neuroplastic change in the brain during vision recovery.

The visual cortex in humans with congenital blindness exhibits decreased functional connectivity (FC) with the frontal motor, parietal somatosensory and temporal multisensory areas [17] and increased FC with the inferior frontal triangular areas [18]. Many studies have shown that the visual cortex dynamically interacts with higher cognitive areas [19, 20]. In PA patients with visual impairment, a researcher identified increased FC between the visual cortex and subareas in the default mode network (DMN) and salience network (SN) [21]. Many of these studies exploring the connection between the vision cortex and other higher cognitive areas mainly focused on persons with early blindness or low vision. No study has reported of this neuroplasticity change in the brain after vision restoration thus far. The changes in the functional connection between the vision cortex and higher cognitive areas in patients with vision recovery remain unclear.

PA may compress the optic apparatus and cause impaired vision. Endoscopic transsphenoidal surgery is minimally invasive and does not damage the visual cortex or neighboring areas. Some patients can experience improved vision rapidly after surgery. During the early period following surgery, however, the change in neurofunction in the extravisual cortex and higher cognitive cortex has yet to be explored. Therefore, we enrolled patients with PA with improved vision at approximately 3 days after a transsphenoidal operation. Our study focused on the changes in the extravisual resting-state networks in patients with PA after vision restoration. Furthermore, we aimed to explore the plasticity of the auditory, language and higher cognitive networks that extend beyond the visual cortex after vision improvement.

## Materials and methods

### Subjects

Fourteen patients with PA with visual damage were recruited in this study. All patients underwent endoscopic transsphenoidal surgery and had improved vision rapidly after the surgery. The inclusion criteria were as follows: age varied between 18 and 65 years; corrected vision acuity was below 1.0 (20/20) before the operation; no ophthalmologic diseases or other intracranial lesions that disturbed the visual apparatus or cortex were found; vision recovery at 3 days after surgery (corrected vision acuity improved by at least 0.2) was needed; and no severe electrolyte imbalance, hypopituitarism or other complications occurred after surgery. This study was approved by the Ethics Committee of the hospital. Written informed consent was obtained from the patients.

### Data acquisition

The patients were scanned one day preoperatively and three days postoperatively on a 1.5 T MR system (Espree, Siemens Medical Solution, Erlangen, Germany) in the diagnostic room of the iMRI brain suite [22]. We used a foam pad to minimize head movement and earplugs to reduce surrounding noise during scanning. During the resting-state functional MRI (RS-fMRI) scan, we told the patients to remain motionless and keep their eyes closed and think about nothing. RS-fMRI data were acquired using an echo-planar image pulse sequence [26 axial slices, slice thickness = 4.5 mm, flip angle = 90°, and field of view (FOV) = 224 × 224 mm, repetition time = 2000 ms and echo time = 45 ms]. A T1-weighted sagittal anatomical image was also obtained using a gradient echo sequence (192 slices, slice thickness = 1 mm, inversion time = 1100 ms, flip angle = 15°, number of excitations = 1, FOV = 256 × 256 mm, repetition time = 1970 ms, and echo time = 2.39 ms).

### Clinical and neuro-ophthalmologic assessments

The cognition of all patients was evaluated with a Mini-Mental State Examination before surgery. The ophthalmologic examination was performed within 2 days prior to the operation and at approximately 3 days after the operation. The best-corrected visual acuity was measured for distance with the E chart and reported on the decimal scale. The ophthalmic fundus examination was made with a nonmydriatic retinal camera (Topcon, Japan).

### Data preprocessing

The RS-fMRI data were preprocessed with SPM8 (http://www.fil.ion.ucl.ac.uk/spm) and a pipeline analysis toolbox, DPARSF (http://www.restfmri.net/) [23]. The first ten volumes were deleted to stabilize the fMRI signal and allow the participants time to adapt to the circumstances. The subsequent data processes consisted of slice timing correction and head motion correction (head motion parameters were < 3 mm in translation and < 3° in rotation). To further reduce the effects of head motion on the estimates of RS activity, we censored volumes within each participant’s fMRI time series that were associated with sudden head motion. For each participant, the fMRI volumes were censored if the framewise displacement of the head position, which was calculated as the sum of the absolute values of the derivatives of the realignment estimates, was greater than 0.5. The data were then normalized (T1-weighted image-based spatial normalization to the Montreal Neurological Institute space). After smoothing, the linear trend of time courses was removed, and then temporal bandpass filtering (0.01–0.08 Hz) was performed.

### FC and statistical analysis

Regions of interest (ROIs) were taken from the literature [24–27]. They were defined as 6-mm radius spheres in both hemispheres. We selected 6 seeds to assess FC (Table 1). These seeds were selected within the extravisual area [auditory cortex (A1), Broca’s area, posterior cingulate cortex (PCC)/precuneus for the DMN, right caudal anterior cingulate cortex for the SN and left dorsolateral prefrontal cortex for the executive control network (ECN)].

**Table 1:** Regions of interest (ROIs)

	Left hemisphere			Right hemisphere			Literature reference
	X	Y	Z	X	Y	Z	
Areas							
A1/BA41	− 42	− 21	7	56	− 13	8	Rademacher et al. [24]
Broca	− 42	26	17				Binkofski et al. [25]
DMN	− 4	− 52	22				Laird et al. [26]
ECN	− 32	41	13				Seeley et al. [27]
SN				4	16	33	Seeley et al. [27]

Before FC calculation, nonneuronal-related covariates, including six parameters of head motion correction, the average time courses of the whole brain (global mean signal), the average time courses within the white matter mask, and the average time courses within the cerebral spinal fluid (CSF) mask, were removed from the preprocessed data by linear regression analysis. Then, the images were smoothed with a 6-mm FWHM Gaussian kernel. We computed the FC between each seed region and each voxel within the whole-brain mask. To improve data normality, the individual FC maps were transformed to z-maps using Fisher’s z-transformation. The z values were entered into a voxelwise paired t test to determine the brain regions that presented significant differences in correlation between the pre- and postoperation groups with separate seed regions. The AlphaSim method, which was implemented in REST, was used to correct for multiple comparisons. The corrected value of p < 0.05 (uncorrected p < 0.001 and a minimum of 40 voxels in a cluster) was chosen as the threshold.

## Results

### Studied population

Fourteen patients (male/female 7:7) were included in the final analyses. The mean age was 46.3 years (range 24–62 years). The main demographic and clinical characteristics of the patients are listed in Table 2.

**Table 2:** The main demographic and clinical characteristics of the patients

No.	Age (Years)	Vision impairment duration (months)	Visual acuity(preop/postop)	
			Left	Right
1	44	6	1/1	0.2/0.5
2	54	2	0.15/0.2	0.1/0.3
3	49	10	0.5/0.8	1/1
4	55	24	0.4/0.6	0.6/0.8
5	46	12	0.2/0.5	0.3/0.4
6	65	1	1/1	0.6/0.8
7	31	2	0.8/0.8	0.4/0.6
8	54	6	0.6/0.9	0.8/0.8
9	46	12	0.3/0.5	0.4/0.5
10	62	12	0.15/0.4	0.8/0.8
11	52	39	0.15/0.4	0.8/0.8
12	40	24	0.5/0.6	0.3/0.5
13	26	0.25	0.8/0.8	0.15/0.4
14	24	24	1/1	0.6/0.9

### RS-fMRI analysis

#### Differences in FC after surgery (auditory ROIs)

**Fig. 1:**
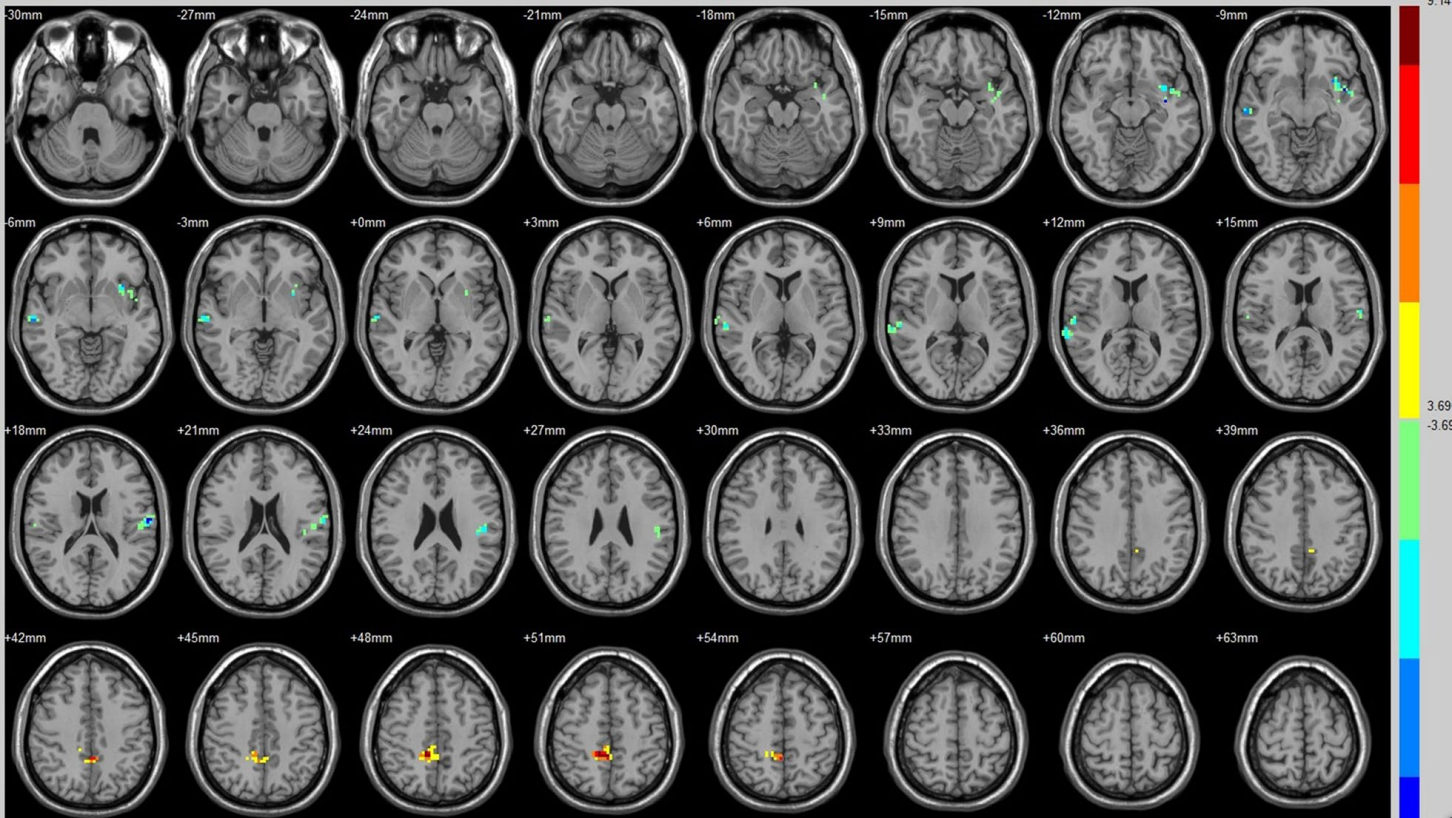
Brain areas exhibited significantly different FCs with the right A1 in PAs (postoperative vs preoperative)

Compared with that of their preoperative counterparts, decreased FC with right A1 was identified in the left insula lobule, right middle temporal gyrus and left postcentral gyrus (Fig. 1, Table 3). Increased FC with right A1 was identified in the right paracentral lobule (Fig. 1, Table 3).

**Table 3:** Group differences in FC (postoperative vs. preoperative)

Seed	Brain region	Peak intensity	Peak MNI coordinate			Cluster size (voxels)
			x	y	z	
Auditory (R)	Insula (L)	− 8.0798	− 36	− 9	− 12	61
	Middle temporal gyrus (R)	− 6.7219	60	− 21	− 9	63
	Postcentral gyrus (L)	− 8.2551	− 60	− 12	18	47
	Paracentral lobule (R)	8.1151	6	− 42	51	80
Broca	Middle temporal gyrus (L)	− 7.8379	− 60	− 12	− 15	56
	Insula (L)	7.3353	− 36	− 12	− 6	56
	Thalamus (R)	8.8303	18	− 12	9	62
DMN	Declive (R)	− 8.282	3	− 78	− 30	73
	Brodmann area 17 (R)	7.976	12	− 105	− 3	45
	Cuneus (L)	6.0238	− 6	− 87	15	49
	Posterior cingulate (R)/BA 30	8.5754	18	− 60	9	51
	Cingulate gyrus (L)	− 12.2874	− 3	− 18	27	48
	Precuneus (R)	− 6.8185	9	− 63	36	43
ECN	Posterior cingulate (R)	− 6.5355	9	− 39	21	46
	Angular (R)	− 8.0772	33	− 57	30	80
	Precuneus (R)	− 5.6647	9	− 48	60	58
SN	Fusiform gyrus (R)	6.9667	33	− 69	− 15	74
	Lingual gyrus (L)/BA 19	5.9544	− 9	− 60	− 3	64
	Middle temporal gyrus (R)	− 6.5879	63	− 39	− 6	44
	Hippocampus (R)	− 7.8909	33	− 18	− 9	92
	Brodmann area 19 (R)	5.9635	42	− 84	3	42
	Corpus callosum (R)	− 7.6753	15	9	24	44
	Precuneus (R)	− 7.239	3	− 63	24	51

#### Differences in FC after surgery (language ROIs)

**Fig. 2:**
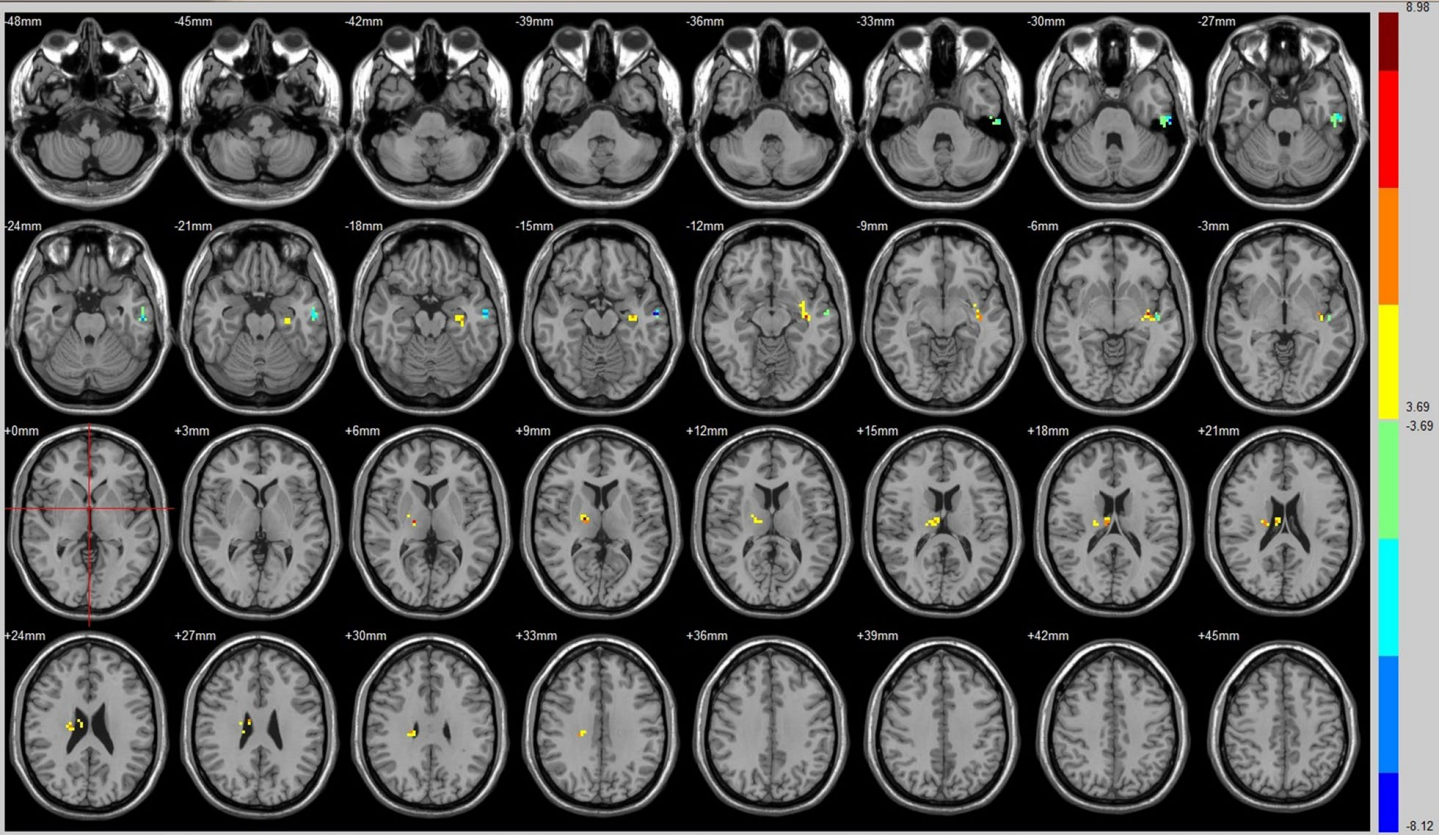
Brain areas exhibited significantly different FCs with the broca in PAs (postoperative vs preoperative)

Compared with that of their preoperative counterparts, decreased FC with the Broca was identified in the left middle temporal gyrus (Fig. 2, Table 3). In addition, compared with preoperative FC, increased FC with the Broca was identified in the left insula lobule and right thalamus (Fig. 2, Table 3).

#### Differences in FC after surgery (DMN ROIs)

**Fig. 3:**
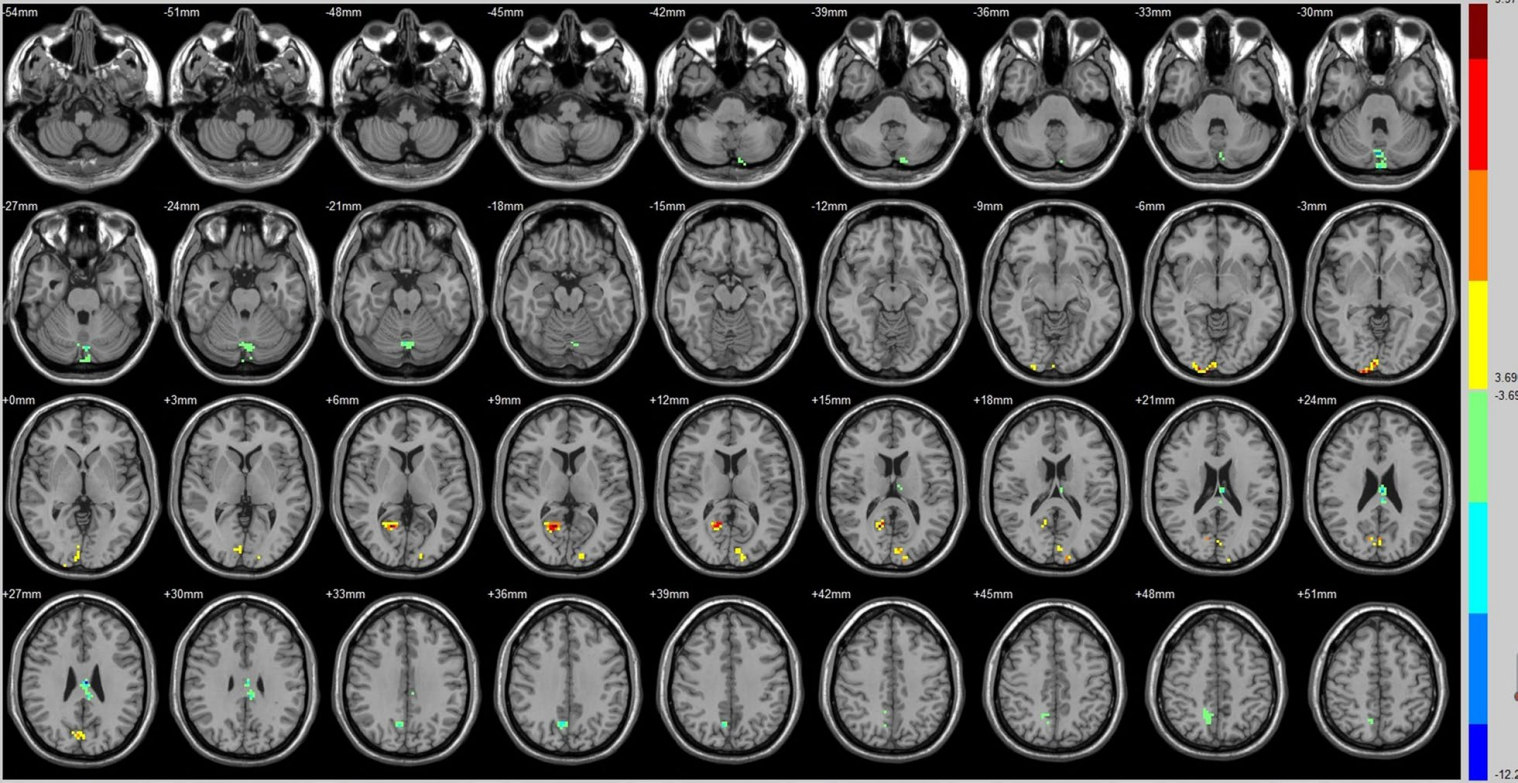
Brain areas exhibited significantly different FCs with the DMN in PAs (postoperative vs preoperative)

Compared with that of their preoperative counterparts, decreased FC with the DMN was identified in the right declive, left cingulate gyrus and right precuneus (Fig. 3, Table 3). additionally, compared with preoperative FC, increased FC with the DMN was identified in right Brodmann area 17, the left cuneus and the right posterior cingulate/BA 30 (Fig. 3, Table 3).

#### Differences in FC after surgery (ECN ROIs)

**Fig. 4:**
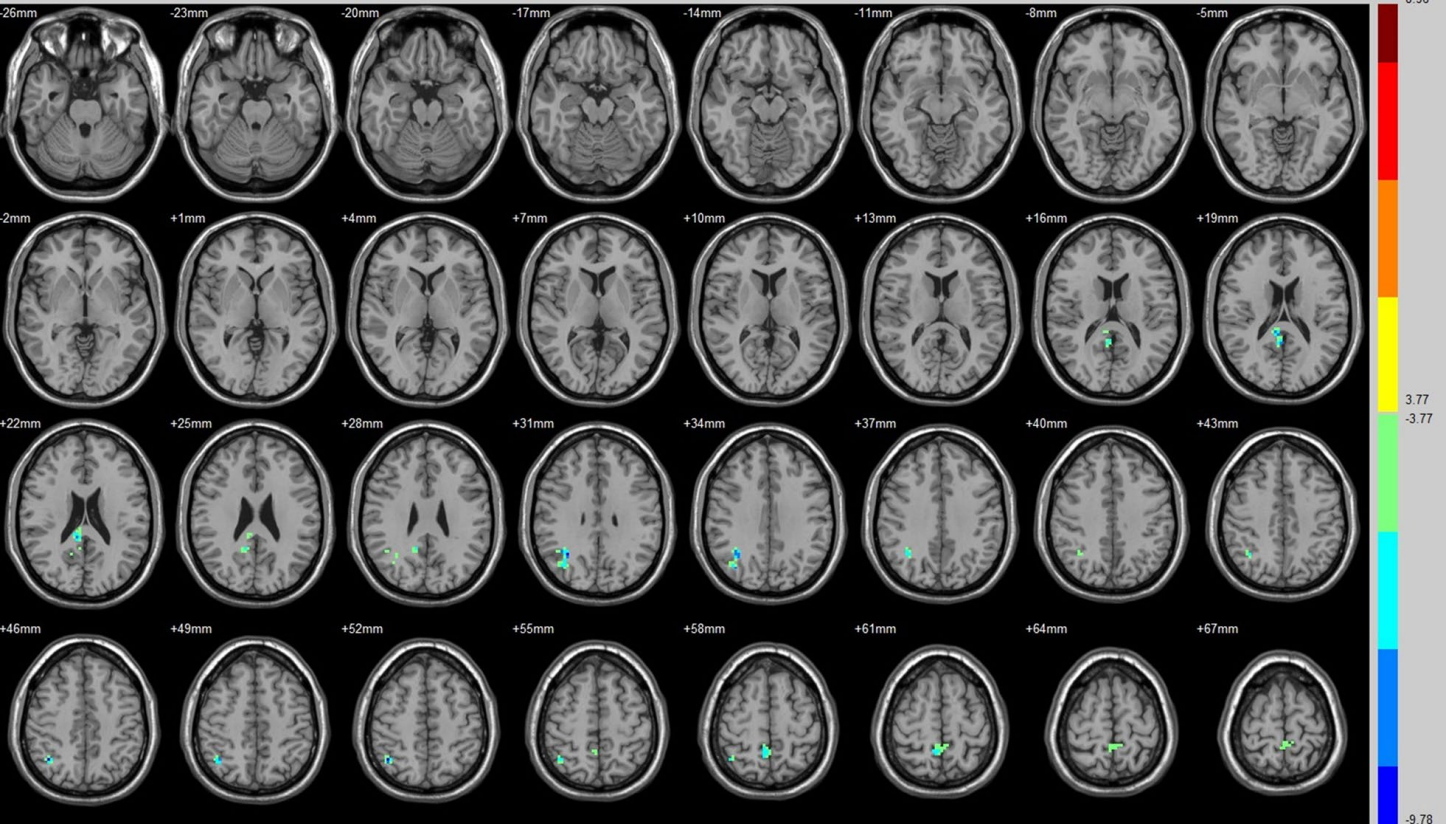
Brain areas exhibited significantly different FCs with the ECN in PAs (postoperative vs preoperative)

Compared with that of their preoperative counterparts, decreased FC with the ECN was identified in the right posterior cingulate, right angular and right precuneus (Fig. 4, Table 3).

#### Differences in FC after surgery (SN ROIs)

**Fig. 5:**
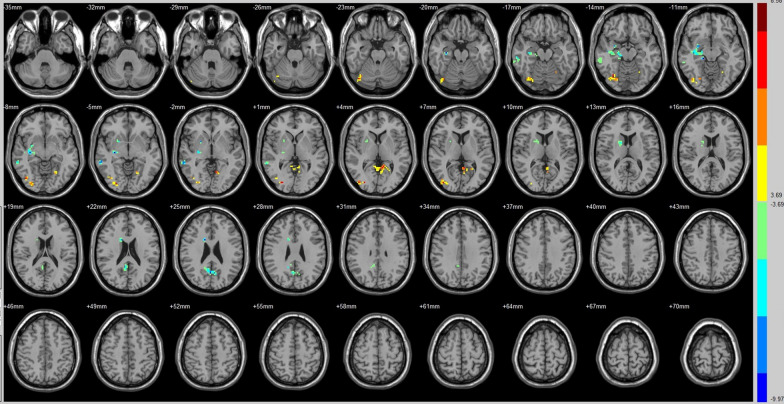
Brain areas exhibited significantly different FCs with the SN in PAs (postoperative vs preoperative)

Compared with that of their preoperative counterparts, decreased FC with SN was identified in the right middle temporal gyrus, right hippocampus, right corpus callosum and right precuneus (Fig. 5, Table 3). Moreover, compared with preoperative FC, increased FC with the SN was identified in the right fusiform gyrus, the left lingual gyrus/BA 19 and right Brodmann area 19 (Fig. 5, Table 3).

## Discussion

RS-fMRI is a noninvasive measure of neuronal function when patients are at rest [28, 29]. FC is widely used in RS-fMRI studies. FC refers to the temporal correlation between spatially remote neurophysiological events [30]. FC analyses can offer high spatial resolution and high spatial specificity relative to where the corresponding changes in neurophysiological signals take place [31]. We investigated FC in extravisual resting-state networks in patients with pituitary adenoma with vision restoration using a seed-based approach with a priori defined ROIs. Our data revealed a mixture of increases and decreases in FC in the brain network.

Our data showed decreased FC with the right A1 in the right middle temporal gyrus (MT). The results may imply that there was a coexisting neural connection between the right A1 and MT, and the FC decreased when vision was restored. The middle temporal complex (MT/MST) is an area specialized for the procession of motion vision [32–35]. Recent studies also show that the visual motion area MT +/V5 responds to auditory motion [36, 37]. In sighted individuals, MT/MST responds to motion perception in the visual modality but not to sound [35]. In contrast, the MT/MST areas of individuals with congenital blindness responds to auditory and tactile motion [38]. Therefore, blindness can cause a multimodal response in the MT/MST region. In individuals who afflicted by early blindness who achieved partial visual restoration in adulthood, auditory motion responses were observed within the MT/V5 area. Therefore, Saenz et al. [36] concluded that auditory and visual responses coexist after vision restoration. Jiang et al. [10] showed that auditory motion responses increased in the MT area and decreased in the right planum temporale in sight-recovery subjects. Therefore, the authors proposed that the cortical plasticity caused by early blindness is permanent and can persist even after visual restoration. Neuroplasticity may have an adaptive or maladaptive effect on the restoration of the deprived sense [9]. Strelnikov et al. [39] showed that synergy between the auditory and visual areas plays a key role in cross-modal plasticity. Alink et al. [40] showed that the auditory motion complex and the visual motion area hMT/V5 + are involved in the generation of a cross-modal dynamic capture illusion and that audiovisual integration occurs in early motion areas. Our results may imply that coexisting neural connections between the right A1 and MT + decreased after vision restoration. To the best of our knowledge, we did not find a possible reason for the decreased FC of the right A1 and MT + , and more fMRI studies are needed in the future.

It is widely accepted that the occipital cortex of humans who are blind is involved in language processing [13]. Braille reading in individuals who are blind triggers a large-scale network of the brain cortex, including posterior and medial occipital areas, fusiform gyrus, area hMT +, inferior temporal gyrus, inferior frontal, prefrontal, intraparietal sulcus, and somatosensory motor areas [41]. In subjects with congenital blindness, the increased connectivity between the visual cortex and Broca’s area might be related to the role of the occipital cortex in semantic processing [13, 42]. Deen et al. [42] proposed that there is coactivation between Broca’s area and most of the occipital cortex. Ricciardi et al. [38] showed that the increased FC between Broca’s area and hMT + might be related to the role of tactile flow processing in Braille reading. There are two explanations for the mechanisms of cross-modal plasticity observed in the occipital cortex. One is that cross-modal plasticity arises through enhancing existing bottom-up sensory connections from sensory areas, and sensory thalamic input during development can reorganize cortical function. The other is that cross-modal plasticity in adults who are blind is activated by top-down feedback from higher-order polymodal and amodal cortices [13, 43]. Our results may imply that FC between the Broca and the left middle temporal gyrus decreased after visual restoration. However, the mechanism resulting in functional alterations in the Broca after vision restoration remains to be elucidated, and more fMRI studies are needed in the future.

Our data show that decreased FC with the right A1 was identified in the left insula lobule and left postcentral gyrus and increased FC with the right A1 was identified in the right paracentral lobule. Increased FC with the Broca was identified in the left insula lobule and right thalamus. The left insula lobule, left postcentral gyrus, right paracentral lobule, and right thalamus are subareas of the multisensory system [44]. The multisensory system at the cortical locations consists of the frontal lobe, temporal lobe, parietal lobe and insula. The multisensory system at subcortical locations involves the superior colliculus and basal ganglia [44, 45]. Insular lesions can result in a multisensory deficiency [46]. The anterior insula has connections with the orbital-frontal lobe, thalamus and limbic lobe. The posterior insula has connections with the frontal, temporal, parietal lobe and thalamus [47, 48]. The thalamus plays a crucial role in multisensory integration processes [49]. It has been accepted that early sensory experience plays an important role in shaping the development of the neural circuitry underlying multisensory processes. In lid-sutured monkeys, studies have shown that visual and multisensory areas become less responsive to visual stimulation [50–52]. In cats reared in darkness, studies have shown that multisensory neurons at cortical and subcortical sites cannot integrate cross-modal inputs [53, 54]. In individuals with congenital dense bilateral cataracts, studies have revealed that their ability to integrate more complex cross-modal stimuli (e.g., speech input) is impaired [55, 56], despite a gain in reaction times for simple cross-modal stimuli (e.g., simultaneously presented light flashes and noise bursts) compared to their unimodal counterparts [57]. Some studies have proposed that superior temporal areas (particularly the superior temporal sulcus) are critical sites for multisensory audio-visual integration [58, 59]. Calvert et al. [58] showed that multisensory audio-visual integration was within extrastriate visual areas. When the brain receives streams of information from multiple sensory modalities, visual information is more frequently preferentially processed than the other sensory modalities. Multisensory information competes for preferential access to consciousness. In terms of multisensory competition, neural representations in the dominant sensory modality may suppress neural representations in the dominated modalities. Weissman et al. [60] reported that enhanced prestimulus activity in the prefrontal cortex and decreased prestimulus activity in the DMN predicted better task performance. Some connectivity studies on visual and auditory activity have shown that sensory systems have dynamic interactions with the prefrontal cortex, the sensorimotor cortex, and the DMN during multisensory competition. The mechanism of multisensory competition is still unclear. One theory that describes this compensation is that top-down control from the prefrontal cortex dominates the outcome of multisensory competition. The other is that the bottom-up processing in the sensory systems decides the outcome. Huang et al. [61] revealed that visual dominance originated from top-down control, while auditory dominance originated from altered sensory processing in the auditory cortex. Our data show that the functional connection between the right A1 and left insula lobule and between the right A1 and left postcentral gyrus decreased and that between the right A1 and right paracentral lobule increased. The functional connection between the Broca and the left insula lobule and right thalamus increased. The study indicates that visual restoration leads to different multisensory interactions within the cortical and subcortical regions, but the mechanism is not clear.

The DMN involves the PCC, medial prefrontal cortex and lateral parietal cortex. The DMN is activated in the resting condition and is deactivated in the task condition. This network plays a role in the detection and monitoring of environmental events and internal mentation [62–64]. Our data show that FC between the DMN and right Brodmann area 17 and the left cuneus increased. When the DMN detects decreased visual cortical activity, decreased deactivation in the DMN may likely occur. Strong DMN activity is related to reduced visual cortical excitability [65]. Our previous data showed that FC decreased in the visual cortex after vision recovery (results not presented in the paper), and this study revealed decreased FC with right A1 and Broca in the right middle temporal gyrus. Therefore, it may be appropriate to propose that decreased visual cortex activity in some way incurs decreased DMN deactivation (stronger activity). It was also assumed that the compensatory mechanism arose as feedback connections by the top-down influences of the DMN. However, the mechanism resulting in functional alterations in the DMN after vision restoration remains to be elucidated. We also found a decrease in the FC between the DMN and the cerebellum (right declive). The cerebellum interacts with the frontal eye fields [66, 67] and participates in the control of eye movement [68, 69]. Our data propose that vision improvement leads to decreased function of the cerebellum with DMN.

The ECN is shown to be activated when fMRI tasks include executive functions. The ECN includes the dorsolateral prefrontal cortex and posterior parietal cortex [70–72]. This network is activated when a task requires cognitive control and working memory [27]. Our data show that decreased FC with the ECN was identified in the right posterior cingulate, right angular and right precuneus after vison restoration. The posterior cingulate/precuneus is a very important part of the DMN. The angular gyrus (AG) plays a role in language and semantic processing [73, 74], spatial attention and orienting [75]. The AG was identified as an important parietal node of the DMN [76–78] and was reported to have task-related deactivations [79]. Therefore, this result may indicate that there is contrary activity in the DMN and CEN after vision recovery. Consistent with our results, some studies show that the DMN and CEN have antagonistic activity in the resting state [72]. Chen et al. [80] found that the ECN has inhibitory control over the DMN. Bauer et al. [81] showed that the ECN negatively regulates the DMN. Whitfield-Gabrieli et al. [82] reported that the anticorrelations between the DMN and ECN are associated with cognitive hyperactivity, such as complex working memory.

The SN constitutes the dorsal anterior cingulate cortex, bilateral insula and presupplementary motor area. This network has a key role in regulating the dynamic changes in other networks. The SN has a function in the commencement of control of cognition processes [83–85]. Our data show that increased FC with the SN was identified in the right fusiform gyrus, the left lingual gyrus/BA 19 and right Brodmann area 19. The results indicate that vision restoration leads to enhanced FC of the visual cortex with the SN. There is an anatomical connection between the visual cortex and the SN [86]. The SN was found to be involved in top-down attentional control [87, 88]. Our previous data showed that FC decreased within the visual cortex after vision recovery (results not presented in the paper). This study revealed decreased FC with the right A1, Broca and SN in the right middle temporal gyrus. Therefore, decreased visual stimulation may result in enhanced FC between the visual system and the SN. Our data show decreased FC with the SN in the right hippocampus, right corpus callosum and right precuneus. The hippocampus is critical for learning, memory and cognition. The hippocampal region has been considered part of the DMN [77, 89]. The precuneus was a key node of the DMN. Our data show that FC between the DMN and right Brodmann area 17 and between the DMN and the left cuneus increased. Studies have reported that the SN drives the DMN during both the resting state and tasks in healthy younger populations [90, 91]. Therefore, the increased activity of the DMN may lead to a decreased influence of the SN on the DMN itself. Taken together, it is reasonable to posit that when decreased FC within the visual cortex is detected, higher than normal SN and DMN activity is initiated to achieve the compensatory mechanism as feedback connection by top-down influences, and then the correspondingly decreased SN activity on DMN occurs in the brain network.

Our data show that decreased FC with the DMN, ECN and SN was simultaneously identified in the right precuneus. The precuneus is an area with high metabolic rates compared to that of other networks during rest. The precuneus is widely accepted as the important structure in the DMN, as it assists in various behavioral functions [92–94]. Many studies have shown that the precuneus plays a vital role in autobiographical memory retrieval, emotional stimulus processing and reward outcome monitoring [95–97]. Some studies have shown that the SN plays a role in the dynamic switching of antagonistic activity between the DMN and CEN in cognitively normal young brains [91, 98, 99]. Multiple experiments have shown that the DMN, SN, and CEN are associated with mindfulness [100–102] and interact with each other [100, 103]. During the active state of meditation, the between-network connectivity of the DMN, CEN and SN is increased [104, 105]. Our results imply that the precuneus may be involved in three networks and relate to the between-network connectivity of the DMN, CEN and SN after visual restoration, but the relevant mechanism is not clear.

Some limitations of this study should be considered. First, the number of subjects in the current study was small, and more participants will be recruited in future studies. Second, most pituitary patients lose visual acuity and fields, and after surgery, the visual acuity and fields may improve. In our study, we only selected visual acuity because it was easy to measure and compare before and after the participants underwent their operation, but it was difficult to quantify the visual field. In future studies, we will evaluate more details on the nature and extent of visual loss and its recovery. Third, testing for changes in visual function with resting-state fMRI may not be the most useful/direct way, but it is convenient to perform in patients pre- and postoperatively. Visual stimuli can be used to directly assess visual responses in fMRI experiments. In future studies, we will combine the two approaches.

## Conclusions

In conclusion, we show the changes in extravisual resting-state networks in patients with pituitary adenoma (PA) with visual restoration after transsphenoidal surgery. Vision restoration may cause a cross-modal plasticity response and lead to the development of the multisensory system related to the A1 and Broca. The DMN and SN may be involved in top-down control of the subareas within the visual cortex. The ECN and SN have decreased FC with the DMN. The precuneus may be involved in the DMN, ECN and SN simultaneously after visual restoration. However, more studies are needed to explore the mechanism of neural plasticity in extravisual resting-state networks, as well as the mechanism of the interaction between the intra- and extravisual networks in patients with specific visual diseases.

## Data Availability

The datasets generated and/or analyzed during the current study are not publicly available due to privacy and ethical restrictions but are available from the corresponding author upon reasonable request.

## Abbreviations

FC: Functional connectivity
A1: Auditory cortex
PCC: Posterior cingulate cortex
DMN: Default mode network
SN: Salience network
ECN: Executive control network
ReHo: Regional homogeneity
PA: Pituitary adenoma
ROIs: Regions of interest
FOV: Field of view
RS-fMRI: Resting-state functional MRI
CSF: Cerebral spinal fluid
MT: Middle temporal gyrus
MT/MST: Middle temporal complex
